# 2-(4-Chloro­phen­yl)-4,5-diphenyl-1-(prop-2-en-1-yl)-1*H*-imidazole

**DOI:** 10.1107/S1600536813012592

**Published:** 2013-05-11

**Authors:** Shaaban K. Mohamed, Mehmet Akkurt, Adel A. E. Marzouk, Francisco Santoyo-Gonzalez, Mahmoud A. A. Elremaily

**Affiliations:** a Chemistry and Environmental Division, Manchester Metropolitan University, Manchester M1 5GD, England; b Chemistry Department, Faculty of Science, Mini University, 61519 El-Minia, Egypt; c Department of Physics, Faculty of Sciences, Erciyes University, 38039 Kayseri, Turkey; d Pharmaceutical Chemistry Department, Faculty of Pharmacy, Al Azhar University, Egypt; e Department of Organic Chemistry, Faculty of Science, Institute of Biotechnology, Granada University, Granada, E-18071, Spain; f Chemistry Department, Faculty of Science, Sohag University, 82524 Shag, Egypt

## Abstract

The title compound, C_24_H_19_ClN_2_, crystallizes with two independent mol­ecules in the asymmetric unit. The prop-2-enyl substituents on the imidazole rings adopt similar conformations in the two mol­ecules. The 4-and 5-substituted phenyl rings and the benzene ring make dihedral angles of 67.06 (8), 5.61 (8) and 41.09 (8)°, respectively, with the imadazole ring in one mol­ecule and 71.53 (8), 28.85 (8) and 41.87 (8)°, respectively, in the other. The crystal structure features C—H⋯π inter­actions and weak π–π stacking inter­actions [centroid–centroid distances = 3.6937 (10) and 4.0232 (10) Å] between the chloro­phenyl rings, which form a three-dimensional supramolecular structure.

## Related literature

For pharmaceutical properties of imidazoles and imidazole-containing compounds, see, for example: Roman *et al.* (2007 ▶); Nanterment *et al.* (2004 ▶); Congiu *et al.* (2008 ▶); Venkatesan *et al.* (2008 ▶); Bhatnagar *et al.* (2011 ▶); Puratchikody & Doble (2007 ▶). For similar structures, see: Mohamed *et al.* (2013 ▶); Akkurt *et al.* (2013 ▶).
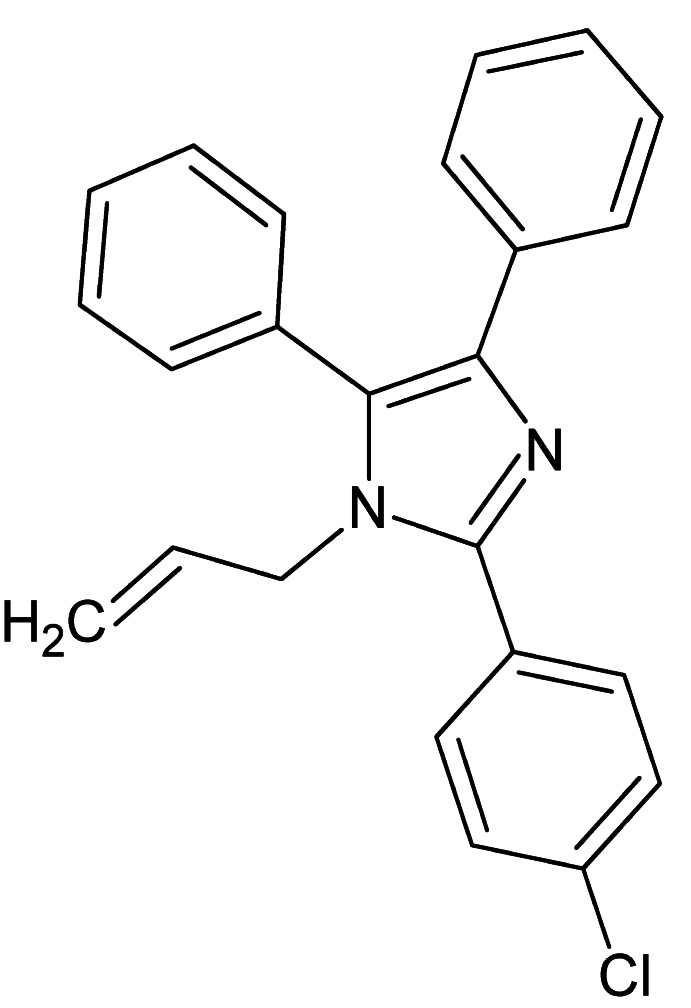


## Experimental

### 

#### Crystal data

C_24_H_19_ClN_2_
*M* *_r_* = 370.86
Triclinic, 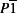

*a* = 10.0916 (7) Å
*b* = 13.1386 (9) Å
*c* = 15.6155 (10) Å
α = 72.924 (1)°
β = 86.849 (1)°
γ = 71.830 (1)°
*V* = 1879.0 (2) Å^3^
*Z* = 4
Mo *K*α radiation
μ = 0.21 mm^−1^
*T* = 100 K
0.57 × 0.33 × 0.28 mm

#### Data collection

Bruker SMART APEX CCD area-detector diffractometer
Absorption correction: multi-scan (*SADABS*; Sheldrick, 2004 ▶) *T*_min_ = 0.919, *T*_max_ = 0.942
20791 measured reflections
7718 independent reflections
6764 reflections with *I* > 2σ(*I*)
*R*_int_ = 0.023

#### Refinement

*R*[*F* ^2^ > 2σ(*F* ^2^)] = 0.041
*wR*(*F* ^2^) = 0.106
*S* = 1.07
7718 reflections
487 parameters
H-atom parameters constrained
Δρ_max_ = 0.34 e Å^−3^
Δρ_min_ = −0.23 e Å^−3^

### 

Data collection: *SMART* (Bruker, 2001 ▶); cell refinement: *SAINT* (Bruker, 2001 ▶); data reduction: *SAINT*; program(s) used to solve structure: *SHELXS97* (Sheldrick, 2008 ▶); program(s) used to refine structure: *SHELXL97* (Sheldrick, 2008 ▶); molecular graphics: *ORTEP-3 for Windows* (Farrugia, 2012 ▶); software used to prepare material for publication: *WinGX* (Farrugia, 2012 ▶) and *PLATON* (Spek, 2009 ▶).

## Supplementary Material

Crystal structure: contains datablock(s) global, I. DOI: 10.1107/S1600536813012592/hg5314sup1.cif

Structure factors: contains datablock(s) I. DOI: 10.1107/S1600536813012592/hg5314Isup2.hkl

Supplementary material file. DOI: 10.1107/S1600536813012592/hg5314Isup3.cml

Additional supplementary materials:  crystallographic information; 3D view; checkCIF report

## Figures and Tables

**Table 1:** Hydrogen-bond geometry (Å, °) *Cg*2, *Cg*4 and *Cg*8 are the centroids of the C4–C9, C19–C24 and C43–C48 rings, respectively.

*D*—H⋯*A*	*D*—H	H⋯*A*	*D*⋯*A*	*D*—H⋯*A*
C5—H5⋯*Cg*4^i^	0.95	2.76	3.5968 (17)	147
C11—H11⋯*Cg*8^ii^	0.95	2.83	3.5879 (19)	137
C33—H33⋯*Cg*2^iii^	0.95	2.89	3.8217 (18)	166

Symmetry codes: (i) 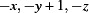; (ii) 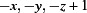; (iii) 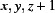.

**Fig. 1.**
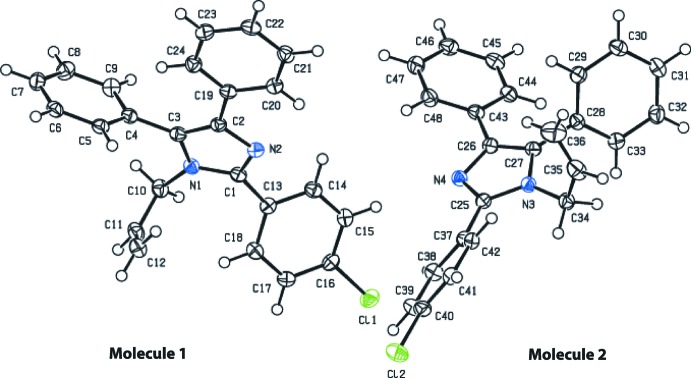
View of the two molecules of the title compound in the asymmetric unit with the atom numbering scheme. Displacement ellipsoids for non-H atoms are drawn at the 50% probability level.

**Fig. 2.**
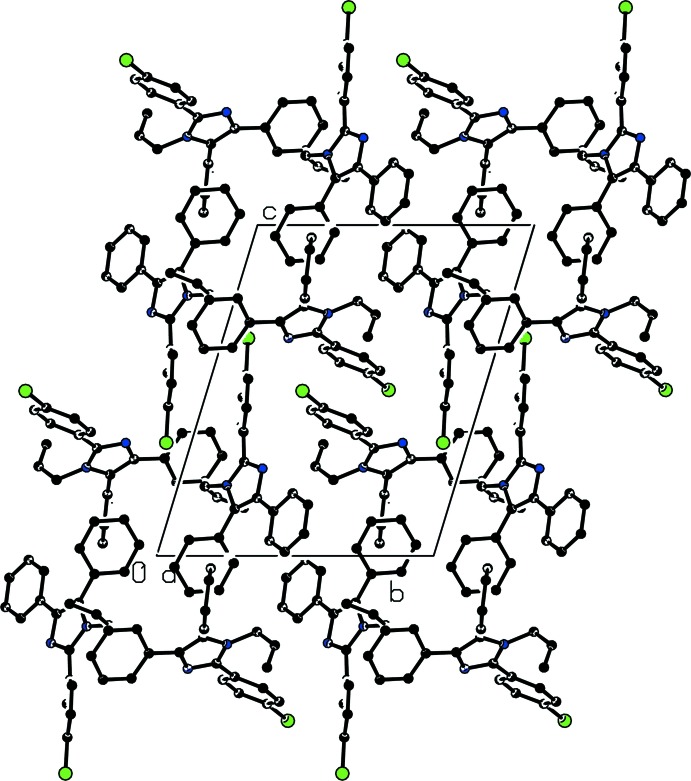
A view of the packing diagram of the title compound viewing along the a axis

## supplementary crystallographic information

### Comment

Imidazoles have been intensivley reported to serve as usefull building blocks for synthesis of diverse class of bioactive molecules. In addition imidazole comtaining compounds exhibited a wide spectrum of pharmaceutical properties such as pesticides, fungicides, antibacterial anti-inflammatory, anti-tubercular, anti-diabetic, antimalarial and antitumour (Roman *et al.*, 2007; Nanterment *et al.*, 2004; Congiu *et al.*, 2008; Venkatesan *et al.*, 2008; Bhatnagar *et al.*, 2011; Puratchikody & Doble 2007). In this aspect and further to our study on synthesis of tetrasubstituted imidazoles as potential bioactive molecules, we herein report the crystal structure of the title compound.

As seen in the Fig. 1, the title compound contains two independent molecules, 1 (with Cl1) and 2 (with Cl2), in the asymmetric unit. The prop-2-ene substituents on the imidazole rings adopt similar conformations in molecules 1 and 2. The 4-and 5-substituted phenyl rings (C4–C9 and C19–C24) and the benzene ring (C13–C18) attached to the atom Cl1 makes dihedral angles of 67.06 (8), 5.61 (8) and 41.09 (8)°, respectively, with the imadazole ring (N1/N2/C1–C3) of molecule 1 and the corresponding angles are A/B = 71.53 (8), A/C = 28.85 (8) and A/D = 41.87 (8)°, respectively, in molecule 2; where A (N3/N4/C25–C27), B (C28–C33), C (C43–C48) and D (C37–C42). All bond lengths are normal and are comparable with those reported for the similar structures (Mohamed *et al.*, 2013; Akkurt *et al.*, 2013).

The crystal structure is stabilized by C—H···π interactions (Table 1) and weak π-π stacking interactions [*Cg*3···*Cg*3 (-*x*, -*y*, 1 - *z*) = 3.6937 (10) Å and *Cg*7···*Cg*7 (-*x*, 1 - *y*, 1 - *z*) = 4.0232 (10) Å; where *Cg*3 and *Cg*7 are the centroids of the C13–C18 and C37–C42 benzene rings respectively, which are attached to the Cl1 and Cl2 atoms]. Fig. 2 shows the packing diagram of (I) along the *a* axis.

### Experimental

The title compound was prepared, according to our reported method (Mohamed *et al.*, 2013) in 81% yield. Suitable single crystals were obtained by slow evaporation of a solution in ethanol, m.p. 305–307 K.

### Refinement

All H atoms were placed in geometrically, with C—H = 0.95 and 0.99 Å, and refined as riding with *U*_iso_(H) = 1.2 *U*_eq_(C) of the parent atom.

### Crystal data

**Table:**

C_24_H_19_ClN_2_	*Z* = 4
*M**_r_* = 370.86	*F*(000) = 776
Triclinic, *P*1	*D*_x_ = 1.311 Mg m^−^^3^
Hall symbol: -P 1	Mo *K*α radiation, λ = 0.71073 Å
*a* = 10.0916 (7) Å	Cell parameters from 9362 reflections
*b* = 13.1386 (9) Å	θ = 4.6–55.6°
*c* = 15.6155 (10) Å	µ = 0.21 mm^−^^1^
α = 72.924 (1)°	*T* = 100 K
β = 86.849 (1)°	Prism, colourless
γ = 71.830 (1)°	0.57 × 0.33 × 0.28 mm
*V* = 1879.0 (2) Å^3^	

### Data collection

**Table:**

Bruker SMART APEX CCD area-detector diffractometer	7718 independent reflections
Radiation source: sealed tube	6764 reflections with *I* > 2σ(*I*)
Graphite monochromator	*R*_int_ = 0.023
phi and ω scans	θ_max_ = 26.5°, θ_min_ = 1.4°
Absorption correction: multi-scan (*SADABS*; Sheldrick, 2004)	*h* = −12→12
*T*_min_ = 0.919, *T*_max_ = 0.942	*k* = −16→16
20791 measured reflections	*l* = −19→19

### Refinement

**Table:**

Refinement on *F*^2^	Primary atom site location: structure-invariant direct methods
Least-squares matrix: full	Secondary atom site location: difference Fourier map
*R*[*F*^2^ > 2σ(*F*^2^)] = 0.041	Hydrogen site location: inferred from neighbouring sites
*wR*(*F*^2^) = 0.106	H-atom parameters constrained
*S* = 1.07	*w* = 1/[σ^2^(*F*_o_^2^) + (0.0514*P*)^2^ + 0.7786*P*] where *P* = (*F*_o_^2^ + 2*F*_c_^2^)/3
7718 reflections	(Δ/σ)_max_ = 0.001
487 parameters	Δρ_max_ = 0.34 e Å^−^^3^
0 restraints	Δρ_min_ = −0.23 e Å^−^^3^

### Special details

**Table:**

Geometry. Bond distances, angles *etc*. have been calculated using the rounded fractional coordinates. All su's are estimated from the variances of the (full) variance-covariance matrix. The cell e.s.d.'s are taken into account in the estimation of distances, angles and torsion angles
Refinement. Refinement on *F*^2^ for ALL reflections except those flagged by the user for potential systematic errors. Weighted *R*-factors *wR* and all goodnesses of fit *S* are based on *F*^2^, conventional *R*-factors *R* are based on *F*, with *F* set to zero for negative *F*^2^. The observed criterion of *F*^2^ > σ(*F*^2^) is used only for calculating -*R*-factor-obs *etc*. and is not relevant to the choice of reflections for refinement. *R*-factors based on *F*^2^ are statistically about twice as large as those based on *F*, and *R*-factors based on ALL data will be even larger.

### Fractional atomic coordinates and isotropic or equivalent isotropic displacement parameters (Å^2^)

**Table:**

	*x*	*y*	*z*	*U*_iso_*/*U*_eq_	
Cl1	−0.20646 (4)	0.09006 (3)	0.65803 (2)	0.0300 (1)	
N1	−0.02163 (12)	0.17529 (10)	0.21321 (8)	0.0200 (3)	
N2	0.06068 (12)	0.28573 (10)	0.26375 (8)	0.0200 (3)	
C1	−0.00889 (14)	0.21232 (12)	0.28437 (10)	0.0197 (4)	
C2	0.09418 (14)	0.29822 (11)	0.17502 (9)	0.0189 (4)	
C3	0.04422 (14)	0.22994 (12)	0.14208 (9)	0.0196 (4)	
C4	0.05483 (15)	0.20489 (12)	0.05485 (10)	0.0203 (4)	
C5	−0.01427 (15)	0.28619 (12)	−0.02220 (10)	0.0218 (4)	
C6	−0.00411 (15)	0.26283 (13)	−0.10377 (10)	0.0233 (4)	
C7	0.07436 (16)	0.15777 (13)	−0.10974 (10)	0.0259 (4)	
C8	0.14332 (17)	0.07618 (13)	−0.03361 (11)	0.0276 (4)	
C9	0.13453 (16)	0.09918 (13)	0.04827 (10)	0.0249 (4)	
C10	−0.09628 (16)	0.09705 (13)	0.20987 (10)	0.0238 (4)	
C11	−0.24612 (17)	0.15345 (14)	0.17687 (10)	0.0277 (5)	
C12	−0.30685 (17)	0.26199 (14)	0.14500 (11)	0.0301 (5)	
C13	−0.06083 (15)	0.17650 (12)	0.37484 (10)	0.0202 (4)	
C14	0.02610 (16)	0.16120 (12)	0.44742 (10)	0.0227 (4)	
C15	−0.01711 (16)	0.13543 (12)	0.53460 (10)	0.0237 (4)	
C16	−0.14858 (16)	0.12304 (12)	0.54890 (10)	0.0222 (4)	
C17	−0.23644 (16)	0.13630 (13)	0.47904 (10)	0.0239 (4)	
C18	−0.19289 (16)	0.16348 (13)	0.39192 (10)	0.0240 (4)	
C19	0.17971 (14)	0.37187 (11)	0.13467 (10)	0.0195 (4)	
C20	0.22749 (15)	0.42241 (12)	0.18880 (10)	0.0221 (4)	
C21	0.31189 (16)	0.48977 (12)	0.15487 (10)	0.0238 (4)	
C22	0.35045 (15)	0.50794 (12)	0.06640 (11)	0.0244 (4)	
C23	0.30485 (16)	0.45768 (13)	0.01221 (10)	0.0249 (5)	
C24	0.22005 (15)	0.39068 (12)	0.04585 (10)	0.0229 (4)	
Cl2	−0.42972 (4)	0.65508 (3)	0.50155 (3)	0.0334 (1)	
N3	0.19935 (12)	0.35109 (10)	0.73274 (8)	0.0194 (3)	
N4	0.17457 (12)	0.23629 (10)	0.65869 (8)	0.0205 (3)	
C25	0.11724 (15)	0.33401 (12)	0.67463 (9)	0.0201 (4)	
C26	0.29983 (15)	0.18823 (12)	0.70807 (9)	0.0197 (4)	
C27	0.31680 (14)	0.25751 (12)	0.75442 (9)	0.0192 (4)	
C28	0.43246 (15)	0.24556 (11)	0.81453 (10)	0.0195 (4)	
C29	0.56009 (16)	0.25167 (13)	0.77852 (10)	0.0236 (4)	
C30	0.67230 (16)	0.23572 (13)	0.83402 (11)	0.0261 (4)	
C31	0.65703 (16)	0.21555 (13)	0.92575 (11)	0.0259 (5)	
C32	0.53069 (17)	0.20896 (13)	0.96205 (10)	0.0269 (4)	
C33	0.41862 (16)	0.22345 (13)	0.90689 (10)	0.0239 (4)	
C34	0.17099 (16)	0.44544 (13)	0.77037 (10)	0.0243 (5)	
C35	0.25101 (17)	0.52512 (13)	0.72946 (12)	0.0310 (5)	
C36	0.33377 (19)	0.52130 (15)	0.66240 (14)	0.0399 (6)	
C37	−0.01782 (15)	0.41483 (12)	0.63418 (9)	0.0202 (4)	
C38	−0.12790 (16)	0.37477 (13)	0.62550 (10)	0.0246 (4)	
C39	−0.25512 (16)	0.44770 (13)	0.58485 (11)	0.0277 (5)	
C40	−0.27089 (15)	0.56125 (13)	0.55261 (10)	0.0245 (4)	
C41	−0.16323 (16)	0.60279 (13)	0.55934 (10)	0.0230 (4)	
C42	−0.03662 (16)	0.52952 (12)	0.60013 (9)	0.0218 (4)	
C43	0.39728 (15)	0.07904 (12)	0.70529 (10)	0.0202 (4)	
C44	0.48893 (15)	0.00912 (12)	0.77813 (10)	0.0222 (4)	
C45	0.58242 (16)	−0.09211 (13)	0.77391 (11)	0.0256 (4)	
C46	0.58688 (16)	−0.12606 (13)	0.69738 (11)	0.0273 (5)	
C47	0.49576 (17)	−0.05782 (13)	0.62534 (11)	0.0279 (5)	
C48	0.40109 (16)	0.04347 (13)	0.62921 (10)	0.0251 (5)	
H5	−0.06880	0.35820	−0.01870	0.0260*	
H6	−0.05110	0.31900	−0.15580	0.0280*	
H7	0.08080	0.14180	−0.16560	0.0310*	
H8	0.19690	0.00410	−0.03750	0.0330*	
H9	0.18270	0.04300	0.10000	0.0300*	
H10A	−0.09340	0.04490	0.27070	0.0290*	
H10B	−0.04730	0.05210	0.17000	0.0290*	
H11	−0.30190	0.10640	0.17930	0.0330*	
H12A	−0.25510	0.31230	0.14130	0.0360*	
H12B	−0.40240	0.29010	0.12560	0.0360*	
H14	0.11660	0.16870	0.43660	0.0270*	
H15	0.04190	0.12650	0.58340	0.0280*	
H17	−0.32590	0.12690	0.49060	0.0290*	
H18	−0.25320	0.17330	0.34350	0.0290*	
H20	0.20190	0.41050	0.24960	0.0260*	
H21	0.34330	0.52350	0.19250	0.0290*	
H22	0.40760	0.55440	0.04310	0.0290*	
H23	0.33170	0.46910	−0.04830	0.0300*	
H24	0.18910	0.35720	0.00790	0.0270*	
H29	0.57050	0.26690	0.71560	0.0280*	
H30	0.75960	0.23860	0.80910	0.0310*	
H31	0.73310	0.20630	0.96360	0.0310*	
H32	0.52050	0.19450	1.02490	0.0320*	
H33	0.33240	0.21830	0.93230	0.0290*	
H34A	0.19370	0.41560	0.83560	0.0290*	
H34B	0.07000	0.48710	0.76200	0.0290*	
H35	0.24060	0.58450	0.75480	0.0370*	
H36A	0.34770	0.46350	0.63470	0.0480*	
H36B	0.38000	0.57630	0.64130	0.0480*	
H38	−0.11570	0.29680	0.64760	0.0290*	
H39	−0.32990	0.42020	0.57930	0.0330*	
H41	−0.17570	0.68070	0.53630	0.0280*	
H42	0.03790	0.55760	0.60490	0.0260*	
H44	0.48700	0.03130	0.83100	0.0270*	
H45	0.64390	−0.13850	0.82380	0.0310*	
H46	0.65140	−0.19510	0.69440	0.0330*	
H47	0.49800	−0.08050	0.57270	0.0330*	
H48	0.33850	0.08880	0.57960	0.0300*	

### Atomic displacement parameters (Å^2^)

**Table:**

	*U*^11^	*U*^22^	*U*^33^	*U*^12^	*U*^13^	*U*^23^
Cl1	0.0315 (2)	0.0358 (2)	0.0215 (2)	−0.0115 (2)	0.0067 (2)	−0.0067 (2)
N1	0.0199 (6)	0.0201 (6)	0.0199 (6)	−0.0079 (5)	−0.0001 (5)	−0.0038 (5)
N2	0.0186 (6)	0.0195 (6)	0.0207 (6)	−0.0055 (5)	0.0020 (5)	−0.0047 (5)
C1	0.0172 (7)	0.0189 (7)	0.0215 (7)	−0.0042 (5)	−0.0004 (5)	−0.0049 (5)
C2	0.0163 (7)	0.0181 (7)	0.0197 (7)	−0.0028 (5)	0.0002 (5)	−0.0044 (5)
C3	0.0173 (7)	0.0189 (7)	0.0201 (7)	−0.0046 (5)	0.0006 (5)	−0.0030 (5)
C4	0.0191 (7)	0.0225 (7)	0.0220 (7)	−0.0102 (6)	0.0018 (5)	−0.0066 (6)
C5	0.0182 (7)	0.0224 (7)	0.0259 (8)	−0.0078 (6)	0.0016 (6)	−0.0074 (6)
C6	0.0204 (7)	0.0293 (8)	0.0210 (7)	−0.0116 (6)	−0.0003 (6)	−0.0041 (6)
C7	0.0292 (8)	0.0322 (8)	0.0227 (7)	−0.0158 (7)	0.0046 (6)	−0.0113 (6)
C8	0.0313 (8)	0.0221 (7)	0.0312 (8)	−0.0082 (6)	0.0049 (7)	−0.0110 (6)
C9	0.0275 (8)	0.0211 (7)	0.0247 (8)	−0.0076 (6)	0.0003 (6)	−0.0043 (6)
C10	0.0278 (8)	0.0232 (7)	0.0236 (7)	−0.0133 (6)	0.0010 (6)	−0.0058 (6)
C11	0.0285 (8)	0.0352 (9)	0.0242 (8)	−0.0179 (7)	−0.0012 (6)	−0.0067 (7)
C12	0.0266 (8)	0.0364 (9)	0.0278 (8)	−0.0096 (7)	−0.0040 (6)	−0.0094 (7)
C13	0.0221 (7)	0.0173 (7)	0.0212 (7)	−0.0064 (6)	0.0014 (6)	−0.0052 (5)
C14	0.0217 (7)	0.0219 (7)	0.0257 (8)	−0.0087 (6)	0.0012 (6)	−0.0065 (6)
C15	0.0258 (8)	0.0238 (7)	0.0226 (7)	−0.0086 (6)	−0.0008 (6)	−0.0072 (6)
C16	0.0270 (8)	0.0191 (7)	0.0198 (7)	−0.0073 (6)	0.0047 (6)	−0.0051 (6)
C17	0.0201 (7)	0.0257 (8)	0.0263 (8)	−0.0087 (6)	0.0038 (6)	−0.0071 (6)
C18	0.0223 (7)	0.0248 (7)	0.0243 (8)	−0.0076 (6)	−0.0017 (6)	−0.0055 (6)
C19	0.0161 (7)	0.0159 (6)	0.0239 (7)	−0.0028 (5)	0.0006 (5)	−0.0044 (5)
C20	0.0212 (7)	0.0217 (7)	0.0227 (7)	−0.0058 (6)	0.0019 (6)	−0.0065 (6)
C21	0.0225 (7)	0.0215 (7)	0.0293 (8)	−0.0072 (6)	−0.0013 (6)	−0.0093 (6)
C22	0.0195 (7)	0.0216 (7)	0.0318 (8)	−0.0092 (6)	0.0037 (6)	−0.0048 (6)
C23	0.0244 (8)	0.0258 (8)	0.0240 (8)	−0.0089 (6)	0.0048 (6)	−0.0059 (6)
C24	0.0240 (8)	0.0229 (7)	0.0232 (7)	−0.0088 (6)	0.0012 (6)	−0.0074 (6)
Cl2	0.0213 (2)	0.0305 (2)	0.0393 (2)	−0.0009 (2)	−0.0062 (2)	−0.0029 (2)
N3	0.0187 (6)	0.0195 (6)	0.0189 (6)	−0.0043 (5)	−0.0008 (5)	−0.0054 (5)
N4	0.0191 (6)	0.0219 (6)	0.0190 (6)	−0.0057 (5)	−0.0006 (5)	−0.0043 (5)
C25	0.0199 (7)	0.0224 (7)	0.0183 (7)	−0.0074 (6)	0.0015 (5)	−0.0056 (6)
C26	0.0192 (7)	0.0203 (7)	0.0176 (7)	−0.0057 (6)	0.0002 (5)	−0.0029 (5)
C27	0.0174 (7)	0.0200 (7)	0.0188 (7)	−0.0053 (5)	0.0012 (5)	−0.0040 (5)
C28	0.0198 (7)	0.0163 (7)	0.0229 (7)	−0.0050 (5)	−0.0019 (5)	−0.0064 (5)
C29	0.0238 (8)	0.0247 (7)	0.0221 (7)	−0.0078 (6)	0.0010 (6)	−0.0062 (6)
C30	0.0210 (7)	0.0277 (8)	0.0311 (8)	−0.0094 (6)	0.0006 (6)	−0.0087 (6)
C31	0.0248 (8)	0.0238 (8)	0.0292 (8)	−0.0074 (6)	−0.0069 (6)	−0.0066 (6)
C32	0.0305 (8)	0.0304 (8)	0.0210 (7)	−0.0111 (7)	−0.0013 (6)	−0.0070 (6)
C33	0.0229 (8)	0.0264 (8)	0.0235 (7)	−0.0085 (6)	0.0022 (6)	−0.0084 (6)
C34	0.0230 (8)	0.0249 (8)	0.0253 (8)	−0.0040 (6)	−0.0018 (6)	−0.0110 (6)
C35	0.0282 (8)	0.0216 (8)	0.0436 (10)	−0.0056 (6)	−0.0063 (7)	−0.0110 (7)
C36	0.0366 (10)	0.0293 (9)	0.0537 (12)	−0.0145 (8)	0.0069 (8)	−0.0083 (8)
C37	0.0198 (7)	0.0236 (7)	0.0163 (7)	−0.0041 (6)	0.0005 (5)	−0.0071 (6)
C38	0.0249 (8)	0.0218 (7)	0.0254 (8)	−0.0066 (6)	0.0000 (6)	−0.0050 (6)
C39	0.0204 (8)	0.0299 (8)	0.0320 (8)	−0.0090 (6)	−0.0023 (6)	−0.0058 (7)
C40	0.0192 (7)	0.0266 (8)	0.0227 (7)	−0.0016 (6)	−0.0013 (6)	−0.0054 (6)
C41	0.0254 (8)	0.0206 (7)	0.0207 (7)	−0.0044 (6)	0.0002 (6)	−0.0055 (6)
C42	0.0228 (7)	0.0244 (7)	0.0197 (7)	−0.0078 (6)	0.0007 (6)	−0.0079 (6)
C43	0.0178 (7)	0.0198 (7)	0.0243 (7)	−0.0080 (6)	0.0014 (6)	−0.0060 (6)
C44	0.0212 (7)	0.0222 (7)	0.0241 (7)	−0.0091 (6)	0.0001 (6)	−0.0053 (6)
C45	0.0203 (7)	0.0231 (7)	0.0303 (8)	−0.0057 (6)	−0.0029 (6)	−0.0038 (6)
C46	0.0218 (8)	0.0217 (7)	0.0380 (9)	−0.0042 (6)	0.0010 (6)	−0.0107 (7)
C47	0.0270 (8)	0.0283 (8)	0.0322 (8)	−0.0075 (7)	0.0004 (6)	−0.0154 (7)
C48	0.0240 (8)	0.0244 (8)	0.0257 (8)	−0.0054 (6)	−0.0039 (6)	−0.0068 (6)

### Geometric parameters (Å, º)

**Table:**

Cl1—C16	1.7458 (16)	C18—H18	0.9500
Cl2—C40	1.7480 (17)	C20—H20	0.9500
N1—C1	1.364 (2)	C21—H21	0.9500
N1—C3	1.3925 (19)	C22—H22	0.9500
N1—C10	1.465 (2)	C23—H23	0.9500
N2—C2	1.3822 (18)	C24—H24	0.9500
N2—C1	1.320 (2)	C25—C37	1.477 (2)
N3—C34	1.467 (2)	C26—C27	1.371 (2)
N3—C27	1.386 (2)	C26—C43	1.476 (2)
N3—C25	1.3706 (19)	C27—C28	1.479 (2)
N4—C25	1.324 (2)	C28—C29	1.393 (2)
N4—C26	1.384 (2)	C28—C33	1.393 (2)
C1—C13	1.475 (2)	C29—C30	1.389 (2)
C2—C19	1.477 (2)	C30—C31	1.387 (2)
C2—C3	1.380 (2)	C31—C32	1.384 (2)
C3—C4	1.483 (2)	C32—C33	1.391 (2)
C4—C5	1.396 (2)	C34—C35	1.495 (2)
C4—C9	1.402 (2)	C35—C36	1.306 (3)
C5—C6	1.387 (2)	C37—C38	1.397 (2)
C6—C7	1.388 (2)	C37—C42	1.397 (2)
C7—C8	1.387 (2)	C38—C39	1.391 (2)
C8—C9	1.390 (2)	C39—C40	1.387 (2)
C10—C11	1.505 (2)	C40—C41	1.380 (2)
C11—C12	1.315 (3)	C41—C42	1.388 (2)
C13—C14	1.400 (2)	C43—C44	1.402 (2)
C13—C18	1.397 (2)	C43—C48	1.393 (2)
C14—C15	1.384 (2)	C44—C45	1.388 (2)
C15—C16	1.385 (2)	C45—C46	1.387 (2)
C16—C17	1.380 (2)	C46—C47	1.386 (2)
C17—C18	1.385 (2)	C47—C48	1.391 (2)
C19—C20	1.401 (2)	C29—H29	0.9500
C19—C24	1.396 (2)	C30—H30	0.9500
C20—C21	1.391 (2)	C31—H31	0.9500
C21—C22	1.386 (2)	C32—H32	0.9500
C22—C23	1.387 (2)	C33—H33	0.9500
C23—C24	1.390 (2)	C34—H34A	0.9900
C5—H5	0.9500	C34—H34B	0.9900
C6—H6	0.9500	C35—H35	0.9500
C7—H7	0.9500	C36—H36A	0.9500
C8—H8	0.9500	C36—H36B	0.9500
C9—H9	0.9500	C38—H38	0.9500
C10—H10A	0.9900	C39—H39	0.9500
C10—H10B	0.9900	C41—H41	0.9500
C11—H11	0.9500	C42—H42	0.9500
C12—H12B	0.9500	C44—H44	0.9500
C12—H12A	0.9500	C45—H45	0.9500
C14—H14	0.9500	C46—H46	0.9500
C15—H15	0.9500	C47—H47	0.9500
C17—H17	0.9500	C48—H48	0.9500
			
C1—N1—C3	107.20 (12)	C24—C23—H23	120.00
C1—N1—C10	126.91 (12)	C22—C23—H23	120.00
C3—N1—C10	125.83 (12)	C19—C24—H24	120.00
C1—N2—C2	106.30 (12)	C23—C24—H24	120.00
C27—N3—C34	124.52 (12)	N3—C25—N4	111.56 (13)
C25—N3—C27	106.76 (12)	N3—C25—C37	124.29 (14)
C25—N3—C34	128.66 (13)	N4—C25—C37	124.15 (13)
C25—N4—C26	105.60 (12)	N4—C26—C27	110.18 (13)
N1—C1—C13	126.68 (14)	N4—C26—C43	121.86 (13)
N2—C1—C13	121.93 (14)	C27—C26—C43	127.93 (14)
N1—C1—N2	111.38 (13)	N3—C27—C26	105.91 (13)
N2—C2—C3	109.72 (12)	N3—C27—C28	123.01 (13)
N2—C2—C19	118.31 (13)	C26—C27—C28	131.07 (14)
C3—C2—C19	131.84 (13)	C27—C28—C29	119.28 (13)
N1—C3—C2	105.40 (12)	C27—C28—C33	121.48 (14)
N1—C3—C4	121.12 (13)	C29—C28—C33	119.18 (14)
C2—C3—C4	133.41 (13)	C28—C29—C30	120.42 (14)
C3—C4—C9	120.62 (13)	C29—C30—C31	120.05 (15)
C5—C4—C9	118.78 (14)	C30—C31—C32	119.84 (15)
C3—C4—C5	120.61 (14)	C31—C32—C33	120.30 (14)
C4—C5—C6	120.60 (15)	C28—C33—C32	120.20 (15)
C5—C6—C7	120.34 (14)	N3—C34—C35	114.07 (13)
C6—C7—C8	119.61 (14)	C34—C35—C36	126.56 (17)
C7—C8—C9	120.41 (16)	C25—C37—C38	119.00 (14)
C4—C9—C8	120.26 (15)	C25—C37—C42	121.96 (14)
N1—C10—C11	113.67 (14)	C38—C37—C42	118.96 (14)
C10—C11—C12	125.92 (17)	C37—C38—C39	120.79 (15)
C1—C13—C18	124.28 (14)	C38—C39—C40	118.82 (15)
C14—C13—C18	118.67 (14)	Cl2—C40—C39	119.90 (13)
C1—C13—C14	116.95 (14)	Cl2—C40—C41	118.58 (13)
C13—C14—C15	121.38 (15)	C39—C40—C41	121.53 (15)
C14—C15—C16	118.29 (15)	C40—C41—C42	119.34 (15)
Cl1—C16—C15	119.59 (12)	C37—C42—C41	120.57 (15)
C15—C16—C17	121.89 (14)	C26—C43—C44	121.28 (13)
Cl1—C16—C17	118.52 (13)	C26—C43—C48	120.42 (14)
C16—C17—C18	119.38 (15)	C44—C43—C48	118.30 (14)
C13—C18—C17	120.39 (15)	C43—C44—C45	120.76 (14)
C20—C19—C24	117.97 (14)	C44—C45—C46	120.53 (15)
C2—C19—C20	118.62 (13)	C45—C46—C47	119.06 (16)
C2—C19—C24	123.36 (13)	C46—C47—C48	120.79 (15)
C19—C20—C21	120.95 (14)	C43—C48—C47	120.55 (15)
C20—C21—C22	120.31 (14)	C28—C29—H29	120.00
C21—C22—C23	119.37 (15)	C30—C29—H29	120.00
C22—C23—C24	120.43 (14)	C29—C30—H30	120.00
C19—C24—C23	120.96 (14)	C31—C30—H30	120.00
C4—C5—H5	120.00	C30—C31—H31	120.00
C6—C5—H5	120.00	C32—C31—H31	120.00
C5—C6—H6	120.00	C31—C32—H32	120.00
C7—C6—H6	120.00	C33—C32—H32	120.00
C8—C7—H7	120.00	C28—C33—H33	120.00
C6—C7—H7	120.00	C32—C33—H33	120.00
C7—C8—H8	120.00	N3—C34—H34A	109.00
C9—C8—H8	120.00	N3—C34—H34B	109.00
C8—C9—H9	120.00	C35—C34—H34A	109.00
C4—C9—H9	120.00	C35—C34—H34B	109.00
C11—C10—H10A	109.00	H34A—C34—H34B	108.00
N1—C10—H10B	109.00	C34—C35—H35	117.00
C11—C10—H10B	109.00	C36—C35—H35	117.00
H10A—C10—H10B	108.00	C35—C36—H36A	120.00
N1—C10—H10A	109.00	C35—C36—H36B	120.00
C10—C11—H11	117.00	H36A—C36—H36B	120.00
C12—C11—H11	117.00	C37—C38—H38	120.00
C11—C12—H12A	120.00	C39—C38—H38	120.00
C11—C12—H12B	120.00	C38—C39—H39	121.00
H12A—C12—H12B	120.00	C40—C39—H39	121.00
C15—C14—H14	119.00	C40—C41—H41	120.00
C13—C14—H14	119.00	C42—C41—H41	120.00
C14—C15—H15	121.00	C37—C42—H42	120.00
C16—C15—H15	121.00	C41—C42—H42	120.00
C18—C17—H17	120.00	C43—C44—H44	120.00
C16—C17—H17	120.00	C45—C44—H44	120.00
C17—C18—H18	120.00	C44—C45—H45	120.00
C13—C18—H18	120.00	C46—C45—H45	120.00
C21—C20—H20	120.00	C45—C46—H46	120.00
C19—C20—H20	120.00	C47—C46—H46	120.00
C20—C21—H21	120.00	C46—C47—H47	120.00
C22—C21—H21	120.00	C48—C47—H47	120.00
C21—C22—H22	120.00	C43—C48—H48	120.00
C23—C22—H22	120.00	C47—C48—H48	120.00
			
C3—N1—C1—N2	−0.36 (17)	C13—C14—C15—C16	1.1 (2)
C10—N1—C1—N2	−177.56 (14)	C14—C15—C16—Cl1	179.79 (12)
C3—N1—C1—C13	−179.04 (15)	C14—C15—C16—C17	−0.4 (2)
C10—N1—C1—C13	3.8 (2)	C15—C16—C17—C18	−0.4 (2)
C1—N1—C3—C4	177.32 (14)	Cl1—C16—C17—C18	179.41 (13)
C1—N1—C10—C11	91.50 (18)	C16—C17—C18—C13	0.5 (2)
C3—N1—C10—C11	−85.20 (18)	C2—C19—C24—C23	−177.66 (15)
C1—N1—C3—C2	0.06 (17)	C20—C19—C24—C23	−0.1 (2)
C10—N1—C3—C4	−5.4 (2)	C24—C19—C20—C21	0.4 (2)
C10—N1—C3—C2	177.30 (14)	C2—C19—C20—C21	178.03 (14)
C2—N2—C1—C13	179.25 (14)	C19—C20—C21—C22	−0.1 (2)
C2—N2—C1—N1	0.49 (17)	C20—C21—C22—C23	−0.4 (2)
C1—N2—C2—C3	−0.45 (17)	C21—C22—C23—C24	0.7 (2)
C1—N2—C2—C19	−176.82 (13)	C22—C23—C24—C19	−0.4 (2)
C25—N3—C27—C28	178.62 (13)	N4—C25—C37—C42	−136.27 (16)
C34—N3—C25—N4	−176.98 (13)	N4—C25—C37—C38	40.4 (2)
C25—N3—C34—C35	−104.98 (18)	N3—C25—C37—C38	−140.18 (15)
C34—N3—C27—C28	−4.0 (2)	N3—C25—C37—C42	43.2 (2)
C25—N3—C27—C26	0.04 (15)	N4—C26—C27—N3	−0.33 (16)
C27—N3—C25—N4	0.27 (16)	C27—C26—C43—C44	29.4 (2)
C27—N3—C25—C37	−179.26 (13)	N4—C26—C43—C48	28.1 (2)
C27—N3—C34—C35	78.23 (18)	C43—C26—C27—N3	177.88 (14)
C34—N3—C27—C26	177.43 (13)	C27—C26—C43—C48	−149.97 (16)
C34—N3—C25—C37	3.5 (2)	N4—C26—C27—C28	−178.74 (14)
C26—N4—C25—C37	179.07 (13)	N4—C26—C43—C44	−152.57 (15)
C26—N4—C25—N3	−0.46 (16)	C43—C26—C27—C28	−0.5 (3)
C25—N4—C26—C27	0.49 (16)	N3—C27—C28—C33	74.0 (2)
C25—N4—C26—C43	−177.85 (13)	N3—C27—C28—C29	−108.89 (17)
N1—C1—C13—C14	139.66 (16)	C26—C27—C28—C29	69.3 (2)
N1—C1—C13—C18	−44.2 (2)	C26—C27—C28—C33	−107.87 (19)
N2—C1—C13—C18	137.26 (17)	C27—C28—C29—C30	−177.11 (15)
N2—C1—C13—C14	−38.9 (2)	C29—C28—C33—C32	0.7 (2)
C3—C2—C19—C24	4.5 (3)	C33—C28—C29—C30	0.1 (2)
N2—C2—C3—N1	0.23 (17)	C27—C28—C33—C32	177.86 (15)
N2—C2—C3—C4	−176.54 (16)	C28—C29—C30—C31	−1.2 (3)
C3—C2—C19—C20	−173.05 (16)	C29—C30—C31—C32	1.4 (3)
N2—C2—C19—C20	2.4 (2)	C30—C31—C32—C33	−0.6 (3)
N2—C2—C19—C24	179.91 (14)	C31—C32—C33—C28	−0.5 (3)
C19—C2—C3—N1	175.95 (15)	N3—C34—C35—C36	4.3 (3)
C19—C2—C3—C4	−0.8 (3)	C25—C37—C38—C39	−177.66 (14)
N1—C3—C4—C9	−65.7 (2)	C42—C37—C38—C39	−0.9 (2)
C2—C3—C4—C5	−69.3 (2)	C25—C37—C42—C41	177.44 (13)
N1—C3—C4—C5	114.35 (17)	C38—C37—C42—C41	0.8 (2)
C2—C3—C4—C9	110.7 (2)	C37—C38—C39—C40	0.3 (2)
C3—C4—C9—C8	179.65 (15)	C38—C39—C40—Cl2	−179.77 (12)
C5—C4—C9—C8	−0.4 (2)	C38—C39—C40—C41	0.6 (2)
C9—C4—C5—C6	−0.1 (2)	Cl2—C40—C41—C42	179.66 (11)
C3—C4—C5—C6	179.87 (15)	C39—C40—C41—C42	−0.7 (2)
C4—C5—C6—C7	0.4 (2)	C40—C41—C42—C37	0.0 (2)
C5—C6—C7—C8	−0.3 (3)	C26—C43—C44—C45	−178.42 (15)
C6—C7—C8—C9	−0.2 (3)	C48—C43—C44—C45	1.0 (2)
C7—C8—C9—C4	0.5 (3)	C26—C43—C48—C47	178.02 (15)
N1—C10—C11—C12	6.4 (2)	C44—C43—C48—C47	−1.4 (2)
C18—C13—C14—C15	−1.0 (2)	C43—C44—C45—C46	0.0 (2)
C14—C13—C18—C17	0.2 (2)	C44—C45—C46—C47	−0.5 (3)
C1—C13—C18—C17	−175.91 (15)	C45—C46—C47—C48	0.1 (3)
C1—C13—C14—C15	175.37 (15)	C46—C47—C48—C43	0.9 (3)

### Hydrogen-bond geometry (Å, º)

Cg2, Cg4 and Cg8 are the centroids of the C4–C9, C19–C24 and C43–C48 rings, respectively.

**Table:**

*D*—H···*A*	*D*—H	H···*A*	*D*···*A*	*D*—H···*A*
C12—H12*A*···N1	0.95	2.55	2.880 (2)	100
C20—H20···N2	0.95	2.44	2.804 (2)	102
C36—H36*A*···N3	0.95	2.56	2.887 (2)	100
C5—H5···*Cg*4^i^	0.95	2.76	3.5968 (17)	147
C11—H11···*Cg*8^ii^	0.95	2.83	3.5879 (19)	137
C33—H33···*Cg*2^iii^	0.95	2.89	3.8217 (18)	166

Symmetry codes: (i) −*x*, −*y*+1, −*z*; (ii) −*x*, −*y*, −*z*+1; (iii) *x*, *y*, *z*+1.
